# Purulent Conjunctivitis in Newly-Born Infants; Methods of Treatment

**Published:** 1864-12

**Authors:** Ernest Hart


### *Purulent Conjunctivitis in Newly-Born Infants; Methods of Treatment.* By Mr. Ernest Hart.

There is no disorder more commonly met with in the range of ophthalmic practice than purulent conjunctivitis in infants. And in hospital practice not only is the frequency of the disease very fully exemplified, but the disastrous consequences of its intensity and of the want of proper attention are witnessed in many cases of total blindness from resulting opaque staphyloma, where the eyeball itself has become involved in the destructive inflammatory action.

The greater number of these cases are due to inoculation by blenorrhagic matter from the mother; but neither on the one hand did every blenorrhagic mother infect her child, nor on the other did the disease arise in children solely from that cause. The opinion lately strongly expressed by Dr. Thomas Ballard, that the undue exposure of newly-born infants to strong light is a plentiful cause of the disease, is fully confirmed. Want of cleanliness is another. Moreover the affection appears, like other forms of conjunctivitis, to be occasionally almost epidemic. The early symptoms of the disease are well known to all practitioners. A little swelling and redness of the lids externally, and of the conjunctiva internally, with a gumming of their edges, and an escape of a lemon-colored fluid from between them, consisting of blood-pigment, serosity and fibrin, diluted with the tears. The swelling and redness increase, and an abundant purulent secretion soon occurs. Sometimes ectropion ensues from the excessive swelling of the palpebral conjunctiva. If the disease be not successfully combatted, corneal complications follow: infiltration at the edge of the cornea, and multiple ulcers; then death of the cornea, sloughing and escape of the lens, and staphyloma. This is the history which may be deciphered in the blinded and staphylomatous eyes of children. But happily these results may be considered as very rare and unlikely to accrue, if proper treatment is adopted in due time. The disorder usually appears on the third or fourth day. It is often sufficient at this time, when the lids have just become stiff and congested, to apply a cold lotion, which is very well borne; to see that the eyes are sponged with tepid water (so that a stream runs under the lids) every hour; and to apply to the edge of the lids with a little brush the following ointment, the object of which is to prevent them from sticking together: citrine ointment, one drachm; olive oil, four drachms; lard to one ounce and a half. At the same time a laxative may be administered. When the symptoms are more strongly marked, and the purulent secretion established, more energetic means must be resorted to. The use of an alum lotion (four grains to the ounce) every hour, applied beneath the lids by a small sponge or syringe, is a very useful measure; and in hospital practice, when you cannot count upon seeing the little patient brought regularly and sufficiently often, and other attentions are commonly wanting, it is found that this treatment, by frequent irrigations with alum-water, yields better results and more rapidly arrests the disorder than any other. Where, however, the mother will give proper attention, and in all private cases, the application of *dilute caustic points* is employed and strongly advised; the application to be made to the palpebral conjunctiva, and followed by refrigerants externally. The *dilute caustic points* are prepared by melting nitrate of silver into stick, with one or two parts of nitrate of potash. Thus the degree of cauterization could be graduated at will. It is only necessary to pass the stick over the palpebral conjunctiva. The lid is to be everted for the purpose, and a little brush, impregnated with a solution of common salt, passed over the surface immediately afterwards, so as to neutralize any excess of caustic; and then the surface should be completely cleansed by the brush dipped in cold water. The effect of these cauterizations is startling in the success which attends them, if conducted with care and thoroughly performed. They need in most cases to be repeated several times, and in severe cases once or twice a day for a time. The inflammation soon assumes a healthy character. It is very rare to see any corneal complications, and the most formidable consequences of the disease are avoided. The instillation of strong nitrate of silver drops, &c., is condemned; radiating incisions of the conjunctiva are rarely necessary and sometimes mischievous; and excision of a portion of the mucous membrane is to be opposed. The application of a strong solution of nitrate of silver, as recommended by Dr. Mackenzie, is a useful resource where the solid dilute caustic points are not at hand, but when they are it is decidedly inferior.

